# Addition of a new three-dimensional adjustable cervical thoracic orthosis to a multi-modal program in the treatment of nonspecific neck pain: study protocol for a randomised pilot trial

**DOI:** 10.1186/s13063-019-3337-0

**Published:** 2019-04-29

**Authors:** Ahmed S. A. Youssef, Nan Xia, Shimaa T. E. Emara, Ibrahim M. Moustafa, Xiaolin Huang

**Affiliations:** 1 Department of Rehabilitation Medicine, Tongji Hospital, Tongji Medical College, Huazhong University of Science and Technology, 1095#, Jiefang Avenue, Wuhan, 430030 Hubei China; 2 Basic science department, Faculty of Physical Therapy, Beni-Suef University, Beni-Suef, Egypt; 3 Umm El Atebaa Hospital, Ministry of Health, Giza, Egypt; 4 Basic science department, Faculty of Physical Therapy, Cairo University, 7-Mohamed Hassan El-Gamel st, Naser City, Cairo 002 Egypt; 5 Department of Physiotherapy, College of Health Sciences, University of Sharjah, Sharjah, UAE

**Keywords:** Neck pain, Ambulatory, Orthotic

## Abstract

**Background:**

Nonspecific neck pain (NSNP) is one of the most common musculoskeletal problems treated by orthopaedic physicians and physiotherapists. Posture has emerged as one of the major risk factors associated with NSNP, but most previous studies ignored correct posturing as an effective treatment. Therefore, one of the major challenges faced by clinicians is how to incorporate 3D posture findings into the treatment plane. The present study will evaluate the feasibility of conducting a larger randomized trial. This pilot study is designed to investigate the hypothesis that a multimodal programme supplemented with the addition of a 3D adjustable cervico thoracic posture corrective orthotic (CTPCO) will yield short- and long-term improvement on NSNP management outcomes.

**Methods/design:**

This pilot, single-blind, randomized controlled trial will divide 24 patients into two groups (study and control) using block randomization. Both groups will receive conventional treatment consisting of a moist hot pack, soft tissue mobilization, manual therapy and therapeutic exercise. The study group will undergo ambulatory mirror-image functional re-training wearing a 3D adjustable CTPCO. The primary outcome is feasibility, including recruitment (e.g., time to complete enrolment, recruitment rate), patient retention and adherence to treatment allocation (e.g., session attendance, home practice, use of non-study treatments). The secondary outcomes used to assess the effectiveness of the treatment will include neck pain (measures using the visual analogue scale (VAS)) and neck disability (measures using the neck disability index (NDI)), among other outcome measures, compared between the experimental and control groups. Three-dimensional posture parameters of head measurements will be provided by a Global Posture System (GPS). The outcome measures for determining the treatment effect will be assessed at three intervals: pre-treatment, after 10 weeks of intervention and after 3 months at follow-up.

**Discussion:**

This randomized controlled pilot trial will inform the design of a future full-scale trial. The outcomes will provide some resources for the incorporation of ambulatory mirror-image functional re-training intervention compared to a control group intervention for neck pain, disability and 3D posture parameters.

**Trial registration:**

Prospectively registered at ClinicalTrials.gov, NCT03331120. Registered on 22 October 2017.

**Electronic supplementary material:**

The online version of this article (10.1186/s13063-019-3337-0) contains supplementary material, which is available to authorized users.

## Background

Nonspecific neck pain (NSNP) is one of the most common musculoskeletal problems treated by orthopaedic physicians and physiotherapists [1, 2]. NSNP has an annual incidence rate of 38 to 73% and a lifetime prevalence of approximately 48%, leading to both economic and social problems [3, 4].

Posture has emerged as a major risk factor associated with NSNP [5–8], but most previous studies have ignored correct posturing as an effective treatment. The few studies that used posture corrective strategies were based on a dated concept that did not incorporate the 3D nature of posture into the treatment strategy [9–12]. Therefore, one of the major challenges faced by clinicians is how to incorporate 3D posture findings into the treatment plan.

Harrison and colleagues [13] reported that posture problems occurred in the head, ribs and pelvis in three dimensions in the form of translations and rotational displacements. Therefore, we should consider three-dimensional postural assessment and correction during the treatment of NSNP to obtain long-lasting effects and prevent the recurrence of neck pain.

Several tools are available for objective postural measurements in clinical use, including simple plumb line measure, photographic techniques [14–16], moiré topography [17] and various computer-assisted methods, such as electro goniometers [18]. These methods are used in clinical assessments, but they have limitations, including the inability to measure neck posture as rotations and translations in six degrees of freedom, as mentioned by Harrison and colleagues [13].

The current study will use a 3D analysis system called the Global Postural System (GPS) [19–21], which is a novel device that investigates all postural variables at once and provides the managing physiotherapist with radiation-free and accurate measurements [16]. This device also provides further information about foot pressure analysis and other 3D features that allow bracing designs to be tailored for each patient [20].

Numerous studies have shown that mirror images in motion exercises, which are prescribed specifically to help normalize the patient’s neuromuscular dysfunction and postural deformation via reflecting the patient’s posture across different planes, are more beneficial than a less personalized programme [22–24].

With these considerations in mind and to integrate the findings of 3D postural assessment into the treatment programme, we designed an adjustable cervico thoracic posture corrective orthotic (CTPCO) to be worn by the patient for a short time. The device has the ability to reflect all transitional displacements and rotational movements of the head. Ambulatory exercises will be performed using a treadmill while the CTPCO holds the patient’s reverse posture.

We designed a randomized two-arm pilot trial to investigate the hypothesis that the addition of a 3D adjustable CTPCO to a multimodal programme will produce short- and long-term improvement effects on NSNP management outcomes (i.e., neck pain, neck disability and 3D posture parameters of the head).

The primary aim of this study is to evaluate the feasibility of conducting a larger randomized trial that considers recruitment, compliance to study protocols and adverse events. The secondary aim is to investigate the effect size of the addition of ambulatory mirror image functional re-training via the wearing of a 3D adjustable CTPCO compared to control group interventions for neck pain, disability and 3D posture parameters.

## Methods/design

### Study design

The study will be a single-blind superiority pilot randomized control trial (RCT) with two parallel groups. The study will be performed according to SPIRIT [25] and good clinical practice guidelines (SPIRIT checklist, Additional file 1). The Ethical Committee of Tongji Hospital, Tongji Medical College, Huazhong University of Science and Technology (HUST), Wuhan, China approved the study protocol (certificate of approval number TJ-IRB20170703), which is prospectively registered at ClinicalTrials.gov (NCT03331120). The study will be performed in the Rehabilitation Department at Tongji Hospital, Affiliated with HUST, China.

## Procedures

Potential patients will be recruited through advertisements in clinical waiting rooms and via mobile patient recruitment applications, like Wechat (Tencent Ltd, Shenzhen, China). Eligible patients will be 17 to 40 years of age with a history of neck pain for longer than 3 months and who are interested in taking part in a clinical trial of physical therapy. No details of radiographic features of the neck region will be mentioned in the advertisements. Volunteers will contact the project coordinator or physician of the rehabilitation clinic and will undergo an initial screening. A clinical and radiological examination will be used to exclude the presence of a specific cause of neck pain.

Patients will complete a written, informed consent form, provide demographic data and complete a survey of patient-reported outcome measures. An outcome assessor will measure the rotational movements and translational displacements of the head in relation to the thoracic region using GPS.

Following the baseline assessment, a research assistant will randomize patients to a study group and control group using sealed, numbered envelopes and a randomisation list generated by the “random integer generator” (https://www.random.org/integers/). The randomisations will be restricted to permuted blocks of different sizes. Each random permuted block will be transferred to a sequence of consecutively numbered, sealed, opaque envelopes for storage in a locked locker until required. As each patient formally enters the trial, the researcher will open the next envelope in the sequence in front of the patient. A blinded investigator will perform all outcome assessments at baseline, after 10 weeks of intervention and after 3 months of follow-up.

## Eligibility

### Inclusion criteria

- Male and female subjects age from 17 to 40 years
- Neck pain with equal or greater than 3/10 on a visual analogue scale (VAS) and pain lasting more than 3 months (chronic neck pain) [26, 27]
- Patients with neck disability; this is defined by a score of at least 5 (on a 50-point scale) on the neck disability index (NDI) [28]
- Patients will be included if they have posture abnormalities by screening test using GPS
- Subjects must be able to continue treatment for 10 weeks and then attend 3-month follow-up
- If patients can accept and sign informed consent form

### Exclusion criteria

If patient report any of the following conditions:

- Neck pain associated with whiplash injuries, medical red flag history (such as tumour, fracture, metabolic diseases, rheumatoid arthritis and osteoporosis) [27].
- Neck pain with cervical radiculopathy or neck pain associated with externalized cervical disc herniation [27]
- Fibromyalgia syndrome; to avoid the similarity of fibromyalgia with a NSNP diagnosis, a physician will use the criteria for the clinical diagnosis of fibromyalgia according to the American College of Rheumatology [29]
- If the patient had previous surgery in the neck area (irrespective of the reason for the operation) [27]
- Neck pain accompanied by vertigo caused by vertebra basilar insufficiency or accompanied by non-cervicogenic headaches [27]
- People will also be excluded if they are undergoing any type of pain treatment or they have psychiatric disorders or other problems that contraindicate the use of the techniques in this study [27]
- If patient has true leg length discrepancy and an associated pathology of upper and lower limbs that may interfere with the global posture (e.g., foot, knee or hip deformities)
- The patients will be unable to attend a 10-week treatment programme and follow-up assessments after 3 months

### Interventions

Twenty-four patients will be randomized into two groups (study and control) using block randomization. Both groups will receive conventional treatment consisting of a moist hot pack, soft tissue mobilization, manual therapy and therapeutic exercises.

**Fig. 1:**
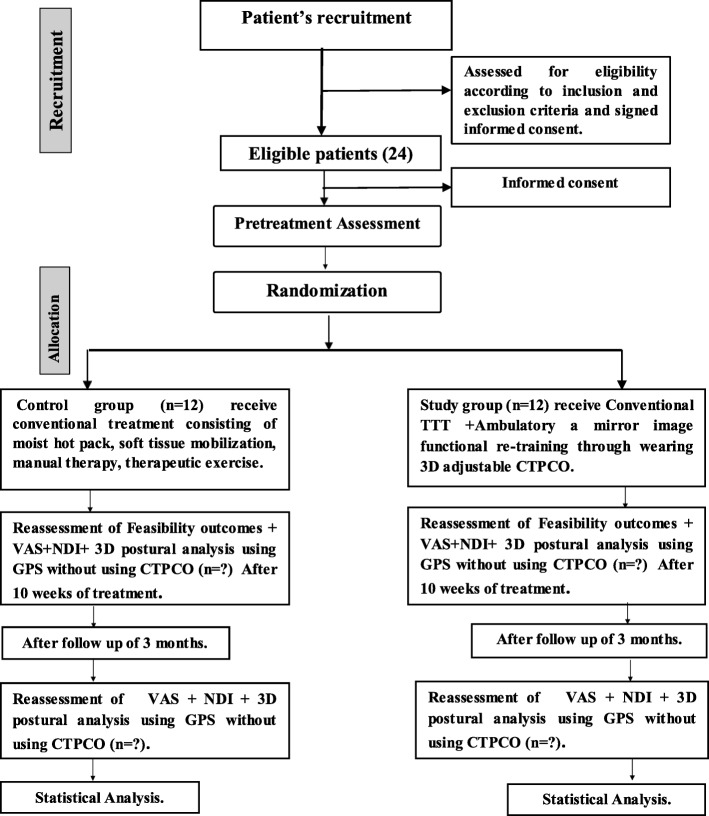
The flow diagram for this trial

The study group will also undergo ambulatory mirror-image functional re-training via the wearing of a 3D adjustable CTPCO. Patients in both groups will attend 30 physical therapy treatment sessions over a 10-week period at three sessions per week and then follow-up after 3 months. Short-term follow-up evaluations will be performed after 10 weeks of interventions, and the long-term follow up will be performed 3 months after the end of the 10 weeks of treatment. The flow diagram for this trial is presented in Fig. 1.

### Conventional treatment

A moist hot pack will be applied to the area of pain in neck region muscles, like the upper part of the trapezius, levator scapulae, splenius capitis and cervicis muscles, for 15 min prior to other conventional treatments to improve the effectiveness of the treatment and reduce short-term pain and disability [30].

Soft tissue mobilization—a deep stroking massage—will be performed along the entire length of the taut band within the following muscles: upper part of the trapezius, supraspinatus, levator scapulae, splenius capitis and cervicis muscles [31].

### Manual therapy

The protocol of Beltran-Alacreu et al. [27] consists of specific passive movements in the facet cervical joints, global mobilization of the cervical spine and a high-velocity technique in the dorsal region. All of these techniques have been proven in previous studies to reduce neck pain and improve joint function of the cervical spine [32, 33].

### Therapeutic exercise

We will create therapeutic exercise programmes for stretching protracted or rounded shoulder muscles and posterior neck muscles in addition to strength exercises of shoulder retractor muscles and the deep cervical flexors, consistent with the protocol described by Harman et al. [12] (Additional file 2).

All of the multimodal programme components will be repeated three times per week for 10 weeks in both groups. All patients will complete the multimodal programme at our physiotherapy clinic.

### Cervico thoracic posture corrective orthotic

**Fig. 2:**
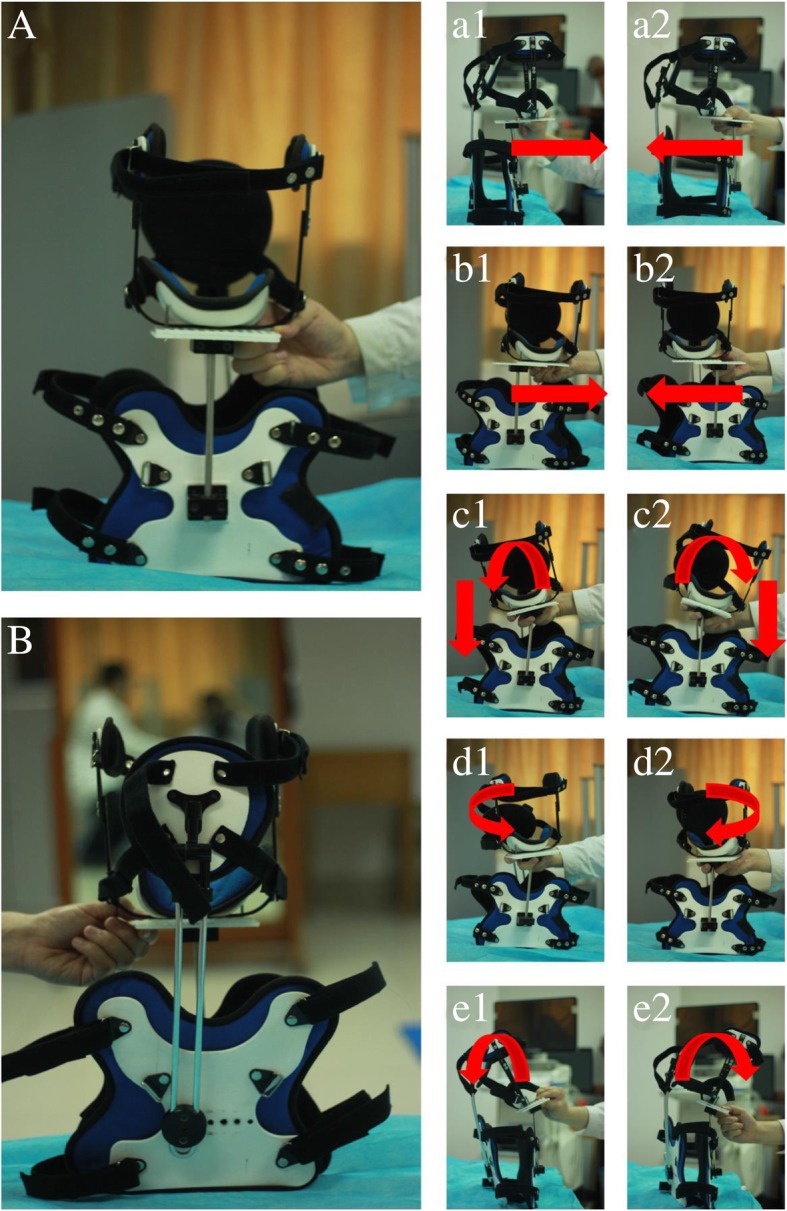
Cervico thoracic posture corrective orthotic (CTPCO). **a** Anterior part of CTPCO. **b** Posterior part of CTPCO. Other images demonstrating the ability to move in different directions: **a1** and **a2** anterior and posterior translation; **b1** and **b2** lateral translation left and right; **c1** and **c2** side bending right and left; **d1** and **d2** rotation left and right; **e1** extension; **e2** flexion

The CTPCO is a low-profile, lightweight, thermoplastic orthotic that is easily applied and removed by the patient. This brace is adjustable and consists of two parts: one part is attached to the thoracic region and is considered a fulcrum on which the other part, which is attached to the head, will be moved. The two parts are connected to each other by a movable joint which allows the movable part to be adjusted in all translational and rotational movements. The brace will reverse (overcorrect) the abnormal posture according to the 3D posture analysis data. The device permits movement in all directions as shown in Fig. 2.

### Ambulatory mirror-image functional re-training via wearing of the 3D adjustable CTPCO

The patient will use the adjustable CTPCO and walk at an approximate speed of 2–3 miles per hour on a standard, motorized treadmill for 20 min, three times per week for 10 weeks. The brace will reverse (overcorrect) the abnormal posture according to the 3D posture analysis data. The facilitation of tissue remodelling using reverse posture training is called mirror-image exercise. An additional movie file shows this procedure in more detail (Additional file 3).


**Additional file 3:** Movie file showing ambulatory mirror image functional re-training through wearing a 3D adjustable CTPCO. (MP4 134029 kb)


The same physiotherapist will individually deliver the entire intervention programme. The physiotherapist has had 10 years of experience and received training in these manual techniques, thereby minimizing inter-therapist variation and increasing reliability.

Patients in both groups will be instructed to perform neck retraction/extension, scapular retraction and deep upper cervical flexor strengthening exercises at home, twice daily as their home routine. To accurately monitor the exercise times and the number of sets performed during the study, a pamphlet illustrating the exercises and a record sheet will be distributed to the patients.

The record sheets will be collected every week and analysed to calculate the mean exercise frequency per week and the mean exercise time per day. The record sheet analysis will reflect a high degree of compliance with the home exercise sessions.

Patients will be encouraged to practice the same home routine at least twice per week for up to three months after treatment, but the ambulatory mirror image functional re-training will be terminated after the initial 10 weeks or 30 visits of intervention. Patients will be contacted via the wechat application every 3 days to gather the record sheet and to inspire patients to continue the training.

### Data collection

The primary outcome in the present study will be measured as the feasibility outcomes of conducting an RCT. The secondary outcome will be measured by the visual analogue scale (VAS), neck disability index (NDI) and the 3D posture parameters measured by the GPS device. The primary and secondary outcomes will be measured at baseline, after 10 weeks and after 3 months of follow-up. The schedule of treatment and outcome assessments is presented in Table 1.

**Table 1:** Schedule of treatments and outcome measures throughout the trial

	Baseline 0 weeks	Treatment period 10 weeks	Follow-up after 3 months
Measures			
Feasibility outcomes	√	√	
VAS	√	√	√
NDI	√	√	√
3D posture parameters	√	√	√
Treatments			
Ambulatory mirror image functional re-training through wearing 3D adjustable CTPCO		√	
Conventional treatment like moist hot pack, soft tissue mobilization, manual therapy, therapeutic exercise		√	

*VAS* visual analogue scale, *NDI* Neck Disability Index, *3D* three-dimensional, *CTPCO* cervico thoracic posture corrective orthotic

### Data collection

In this study, the primary outcome will be measured by feasibility outcomes of conducting an RCT. The secondary outcome will be measured by the Visual Analogue Scale (VAS), Neck Disability Index (NDI) and Three-dimensional posture parameters measured by GPS device. Both the primary and secondary outcomes will be measured at baseline, after 10 weeks and after 3 months of follow-up. The schedule of treatment and outcome assessments is presented in Table 1.

## Primary outcomes

The primary outcome of the study is the feasibility of conducting an RCT [34, 35]. The specific aspects of feasibility that will be monitored are listed below.

### Integrity of the study protocol

Integrity includes the appropriateness of inclusion criteria, training of the staff, clinic accessibility for patients, acceptability of the intervention to patients and physical therapists and the time required for patients and facilities to deliver the interventions. These data will be gathered from interviews with patients and practitioners/therapists on their willingness to participate in the study and their opinions on the research process.

### Recruitment and retention

Recruitment and retention data include procedures for patient enrolment (goal of at least 80% of eligible patients accepting to be enrolled), patient adherence to the intervention (goal of at least 80% of patients attending 75% of treatment sessions and completing 75% of the prescribed exercises) and patient losses to follow-up (goal of at least 80% of participants completing the follow-up) [36].

### Outcome measures

Questionnaires, physical impairment measures and methods to measure exercise intervention compliance (measured via exercise diaries) will be used to determine the completeness of outcome data collected.

### Randomization procedure

The appropriateness of the methods used to ensure the blinding of the outcome measurement assessor will be determined through post-study interviews that ask whether they were aware of the group allocation and whether they felt that the treatments were consistent.

### Primary outcome measure

Selection of the most appropriate primary outcome measure for a full-scale RCT will be determined using the patient-reported outcome measure with the largest between-group effect size, as long as the between-group difference is greater than the previously reported minimal important change for that outcome measure.

### Sample-size calculation

The sample size of a future, fully powered study will be estimated using sample-size calculations using the effect-size data from this pilot study. We will recruit 24 patients for two groups [37].

## Secondary outcomes

### Visual analogue scale

Patients will be asked to indicate their perception of pain along a 10-cm line, with 0 (no pain) on one end and 10 (worst pain) on the other end. Patients will be asked to place a mark along the line to denote their level of pain [38]. The time frame will be pre-treatment, post-treatment 10 weeks and after 3 months of follow-up.

### Neck Disability Index

**Fig. 3:**
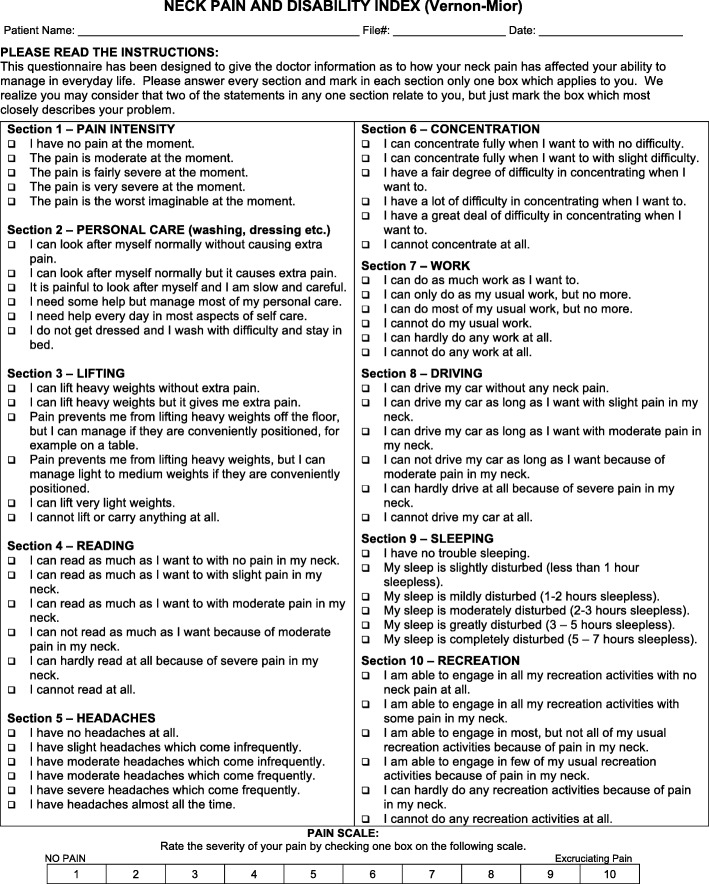
Neck disability index (NDI). English and Chinese versions of the NDI

The NDI is a modification of the Oswestry Low Back Pain Disability Index. It is a patient-completed, condition-specific functional status questionnaire with ten items, including pain, personal care, lifting, reading, headaches, concentration, work, driving, sleeping and recreation. The NDI has sufficient support and usefulness to retain its current status as the most commonly used self-report measure for neck pain. It may be scored as a raw score or doubled and expressed as a percentage. Each section is scored on a 0 to 5 rating scale, in which zero means ‘no pain’ and 5 means ‘worst imaginable pain’. All of the points may be summed to a total score. The test may be interpreted as a raw score, with a maximum score of 50, or as a percentage. Zero points or 0% means no activity limitations, and 50 points or 100% means complete activity limitation (English and Chinese versions of the NDI are shown in Fig. 3) [28]. The time frame will be pre-treatment, post-treatment 10 weeks and after 3 months of follow-up.

### Three-dimensional posture parameters of the head region in relation to the thoracic region

#### Instrumentation for measurement

**Fig. 4:**
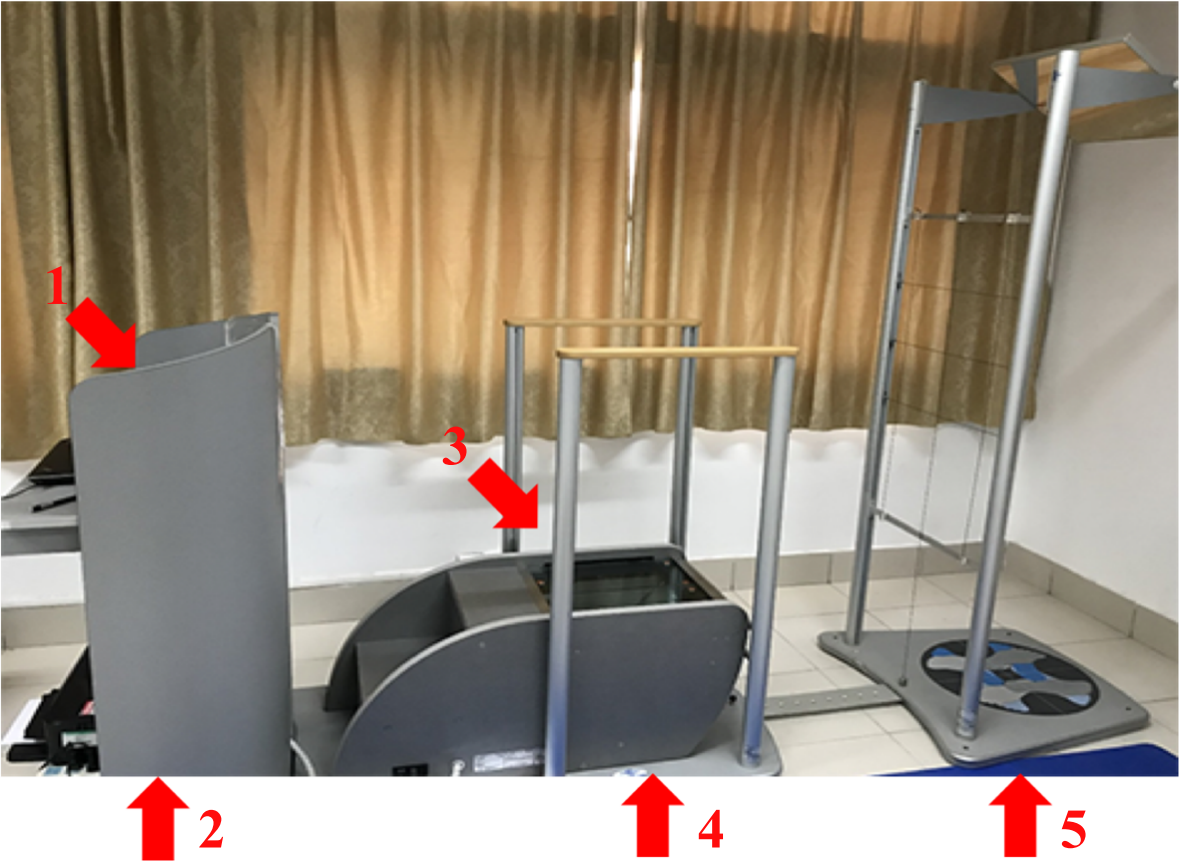
Global posture system (GPS). The GPS 600 device (Chinesport, Udine, Italy) consists of *1* software GPS 5.0, *2* desktop, *3* podostabil, *4* podata, *5* Lux postural analyzer

For assessment purposes, the Global Posture System (GPS) 600 (Chinesport, Udine, Italy), as shown in Fig. 4, is a novel and unique device that is used to examine all postural variables at once [19–21]. This posture analysis system consists of a number of units and software that make it possible to acquire images for body part measurements and collect information on weight distribution, barycentre and the stability of the patient being examined; it will be used per the manufacturer’s instructions [19]. We will analyse the posture of the head in relation to the thoracic region in terms of translations and rotations.

#### Components of GPS

1. Software GPS 5.0
2. Desk top
3. Podostabil
4. Podata
5. Lux postural analyzer

### Assessment procedures

#### Assessment location

Any room in any place or clinic may be used for the application of GPS. No preparations are needed for the room, but the study room will have sufficient space and flat, plain-coloured walls.

#### GPS setup and operation

The GPS will be calibrated prior to measurement.

#### Preparation of patients

Patients will be asked to wear tight-fitting clothes to allow the examiners to find various anatomical sites. The examiners will place 13 markers on each patient before taking four photographs.

#### Marker placement

**Fig. 5:**
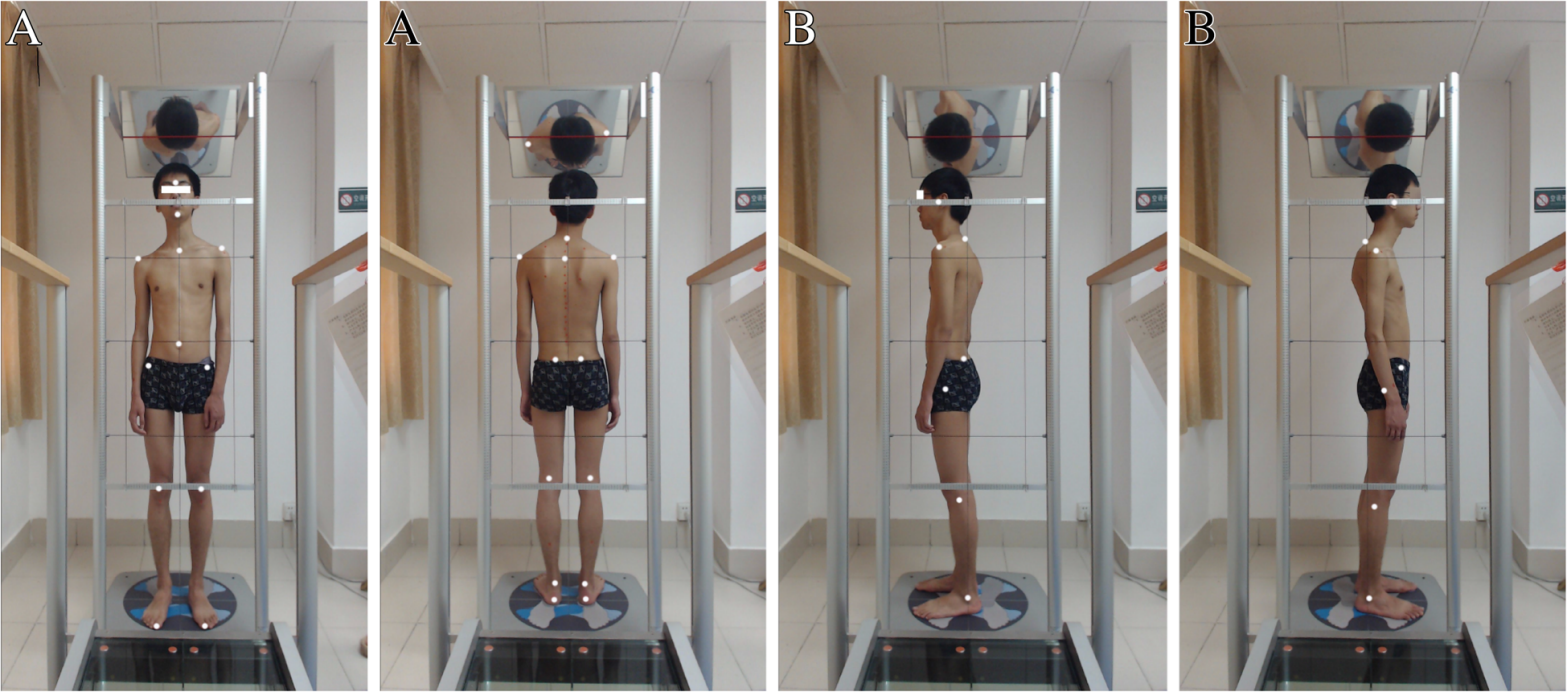
Examples of the photographs taken using the Global Posture System (GPS). **a** Anterior and posterior views. **b** Sagittal plane or lateral views. The six reflective markers used in the analysis are: acromion, anterior superior iliac spine, posterior superior iliac spine, glabella, tragus, C7 and middle sternal notch

1. Antero posterior view markers. Thirteen coloured markers at anatomical locations as shown in Fig. 5.
2. Two lateral view markers. Thirteen coloured markers at anatomical locations as shown in Fig. 5. The points over which the markers are fixed are well cleaned with alcohol to remove sweat and ensure good fixation. Four photographs or four views will be obtained for every patient: anterior and posterior views and two lateral (right and left) views.

#### Starting position of the patients

For the photographs, patients will be instructed to stand on the Lux postural analyzer part of the GPS, take a deep breath three to five times for full relaxation, nod their head up and down twice with their eyes closed and assume what they feel to be a neutral body posture. Their eyes will be open and the subject stopped from moving during this stance. Four digital photographs will be taken using a computer mouse. The set of photographs will be processed through secure software analyses using GPS.

### Measured items (the postural parameters) of the head region in relation to the thoracic region

**Fig. 6:**
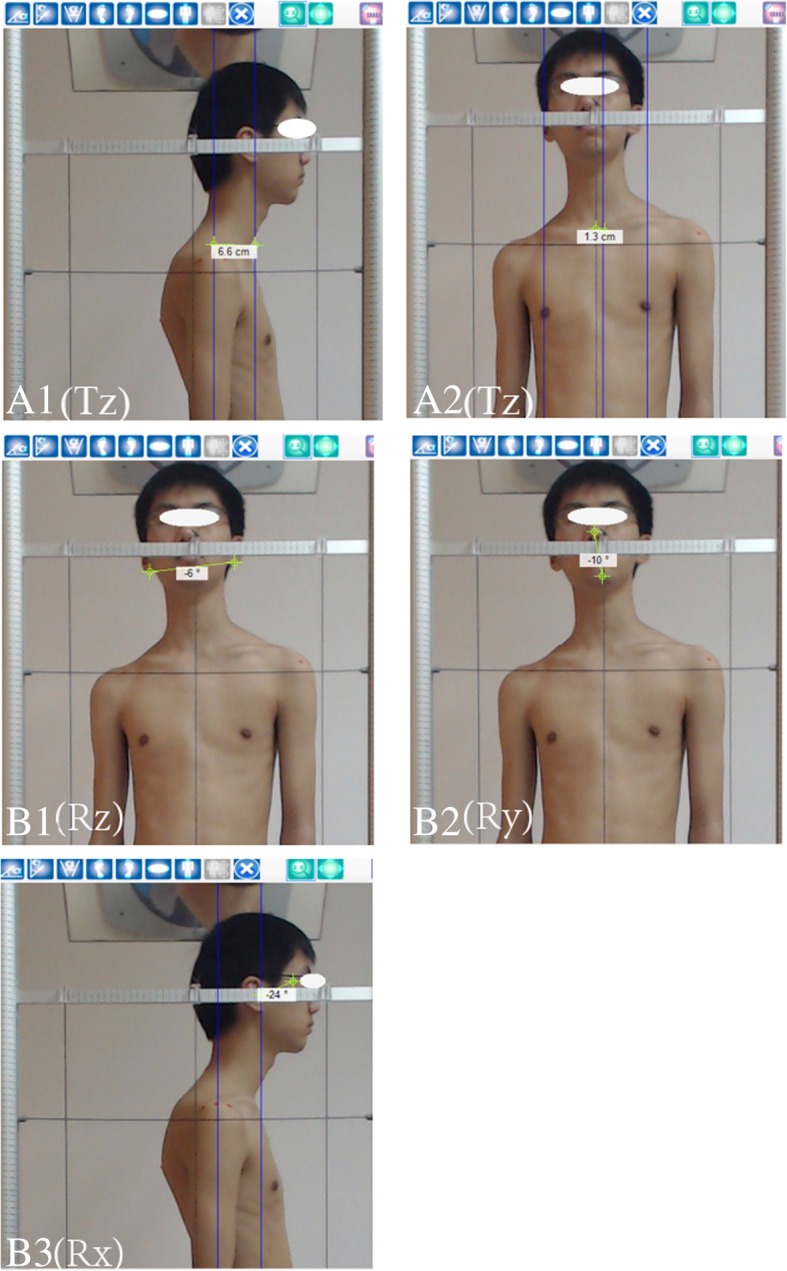
Three-dimensional posture parameters measured by the GPS device. The postural parameters of the head in relation to the thoracic region: Postural translation A1 (Tz), A2 (Tx) measured in centimetres. Postural rotations B1 (RZ), B2 (Ry), B3 (Rx) measured in degrees

A right-handed Cartesian coordinate system with x-axis positive to the left, y-axis positive vertically and z-axis positive to the anterior will be used to describe postures of the head as translations or displacements in centimetres (Tx, Ty, Tz) along these axes and as rotations (Rx, Ry, Rz) in degrees from a normal upright stance. Vertical translations (Ty), which would require radiographic analysis of hypo- or hyper-lordosis, will not be calculated in the present study (Fig. 6) [13].

### Postural translations of the head

1. Tx (right or left side shifting or lateral translation): measure horizontal distance from the ideal plumb line passing through middle sternal notch to vertical line passing through nose.
2. Tz (anterior or posterior translation): measure horizontal distance from the vertical line crossing the middle acromion process to the vertical line crossing the external ear.

### Postural rotations of head

1. Rx (flexion or extension position): measure angle between tragus of ear, canthus of eye and the horizontal line.
2. Ry (right or left rotation): measure angle between glabella of forehead or tip of nose, the middle point of chin and the vertical line.
3. RZ (right or left side bending): measure angle between the inferior margins of the right and the left ear and the horizontal line.

The time frame is pretreatment, post-treatment 10 weeks and after 3 months of follow-up.

### Withdrawal and dropout

All patients will have the right to withdraw from the study at any time. Participation will be terminated at any stage if the patient refuses to continue, withdraws their consent or violates the inclusion or exclusion criteria or the trial protocol. The trial will be terminated if the principal investigator believes that there are unacceptable risks of serious adverse events.

### Statistical analysis

Results of primary outcome measures will be provided descriptively in tables and compared to the a priori established goals. To provide a recommendation for estimated sample size for a future full-scale RCT, between-group effect sizes and 95% confidence intervals (CIs) with Hedges correction will be calculated for changes in the secondary outcomes of VAS, NDI and 3D posture parameters. The mean ± standard deviation score for each of the subscales will be used in the calculation of the effect size. Estimated sample size will be determined using the between-subject effect size, with a minimum of 80% power (*α* = 0.05). The sample size will be increased to allow for an estimated 20% dropout rate. Statistical methods for the secondary outcome measures will be evaluated via comparisons of changes between groups. Complete case analyses will be performed to include outcomes from all patients who completed baseline and follow-up evaluations, as recommended in the Consolidated Standards of Reporting Trials (CONSORT) guidelines [39]. The between-group difference in change scores for each outcome measure from baseline to follow-up will be determined and reported as the means and 95% CI, using an analysis of covariance. Covariates of age and sex will be included in the analysis. All statistical analyses will be performed in SPSS version 23.0 software (IBM Corporation, Armonk, NY) with significance set at *P* < 0.05.

## Discussion

NSNP is one of the four most frequently reported musculoskeletal problems [40]. It is predictable in the adult ‘world population’, which exhibits a mean lifetime incidence of 48%, annual incidence of 38 to 73%, monthly incidence of 25%, one week prevalence ranging from 8 to 45%, and point incidence of 10% [4]. Approximately one-fifth of adults who were previously pain-free report a new episode of neck pain in a 1-year period [41].

NSNP is a frequent complaint. It is a recognized as a medical and socioeconomic problem and is a frequent cause of employment termination worldwide [40].

Although the pathoanatomical cause of NSNP is not known [40], a significantly higher incidence of pain was found in subjects with more severe postural abnormalities. Therefore, further research is necessary based on these findings.

There is strong relationship between cervical posture abnormalities, neck pain and disability in patients, especially in those aged 20 to 50 years [5–8]. However, treatment programmes do not depend primarily on 3D posture assessments and corrections to treat the cause and prevent recurrence of neck pain [9–12].

Therefore, this randomized pilot trial will inform the design of future full-scale trials. The outcomes will provide some resources for the integration of an ambulatory mirror-image functional re-training intervention compared to a control group intervention for neck pain, disability and 3D pasture parameters.

Improvement in the current study in the postural parameters in terms of translational displacements and rotational movements in six degrees of freedom will likely occur for various reasons.

The first reason is that the corrective bracing protocol in the current study will be tailored to each patient according to the 3D postural analysis. The protocol addresses the neuromuscular and skeletal factors involved in the progression of postural deformity [24].

The second reason to expect more effective changes is because the treatment programme in this study will concentrate on rehabilitation of the spine in a reflexive environment. This interpretation agrees with Christensen, who reported the important role of rehabilitation in a reflexive environment, especially for posture correction exercises because posture is highly controlled by reflex activity [42]. The third reason to expect a more effective change is because moving spinal tissues elongate and remodel more effectively than static spinal tissues.

### Limitations of this study

The major limitation of this protocol is its non-double-blind design. However, the outcome assessors and statistical analysts will be blinded to the intervention to decrease potential bias and ensure the prominence of this trial. This study also lacks long-term follow-up observations and assessments. The follow-up evaluation will occur 3 months after completion of the 10-week intervention period, which will be an effective evaluation of short and medium periods for almost 6 months. In conclusion, the results of this study are expected to provide information on the feasibility of conducting RCT evaluations of the effect of the addition of a new, 3D adjustable CTPCO to multimodal treatment of NSNP and evaluations of the use of ambulatory mirror-image functional re-training via the wearing of a CTPCO as one of the multimodal exercises used for correcting posture in 3D directions in NSNP patients.

### Trial status

Ongoing recruitment.

## Additional files

Additional file 1: SPIRIT 2013 checklist. (DOC 121 kb)

Additional file 2: Conventional treatment. (PDF 628 kb)

## Abbreviations

3D: Three-dimensional
ASIS: Anterior superior iliac spine
CONSORT: Consolidated Standards of Reporting Trials
CTPCO: Cervico thoracic posture corrective orthosis
GPS: Global Postural System
HUST: Huazhong University of Science and Technology
NDI: Neck disability index
NSNP: Nonspecific neck pain
PSIS: Posterior superior iliac spine
RCT: Randomised control trial
VAS: Visual analogue scale
