# Supplementary material for: Addition of a new three-dimensional adjustable cervical thoracic orthosis to a multi-modal program in the treatment of nonspecific neck pain: study protocol for a randomised pilot trial
Source: Trials. 2019 Apr 29;20:248. doi: 10.1186/s13063-019-3337-0 (PMC6489278; doi:10.1186/s13063-019-3337-0)

Supplemental Digital Content

1-Moist hot pack: on area of pain at neck region muscles like upper part of trapezius, levator scapulae, splenius capitis, and cervicis muscles for 15 minutes.

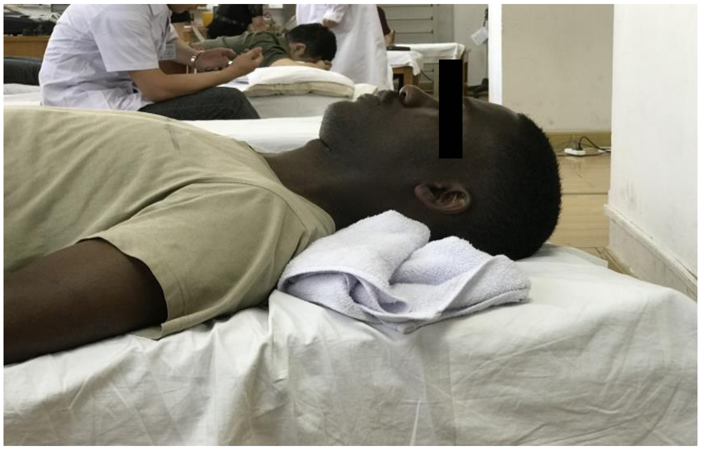

2-Soft tissue mobilization Deep stroking massage will be performed along the entire length of the taut band within the following muscles: upper part of trapezius, supraspinatus, levator scapulae, and splenius capitis and cervicis muscles.

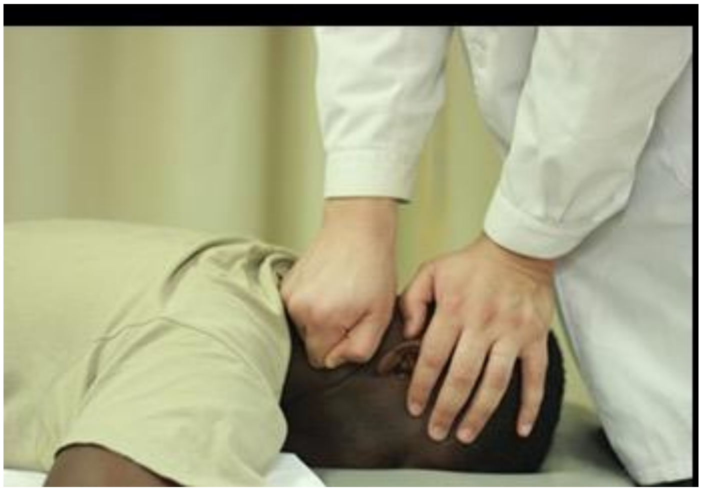

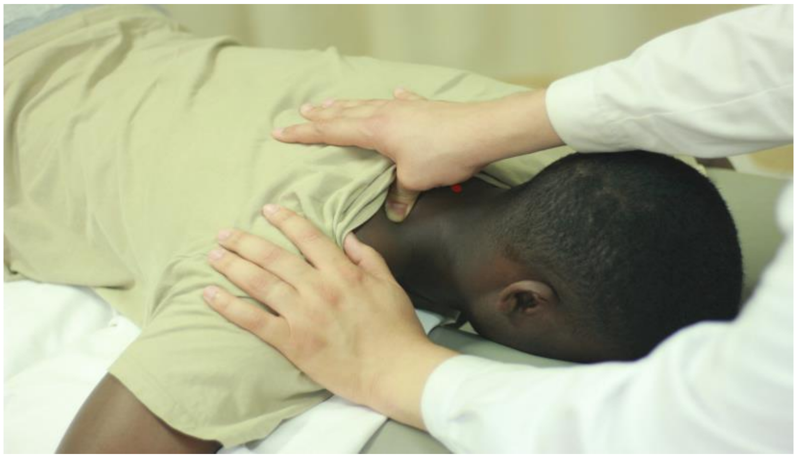

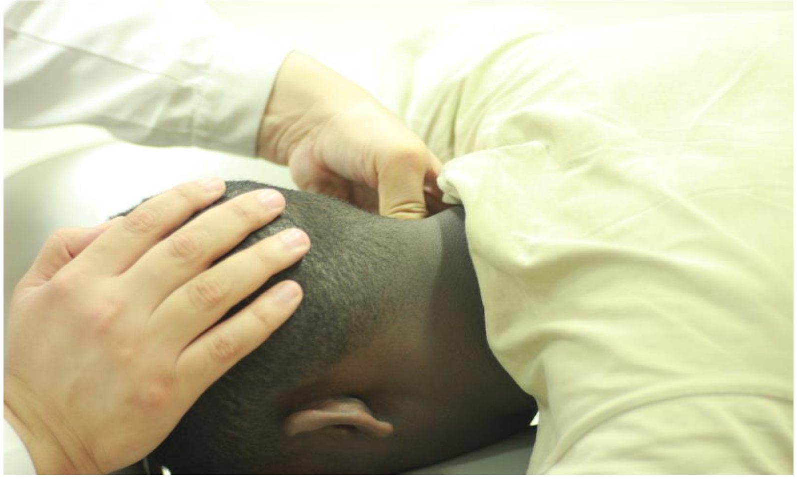

3. Manual Therapy Techniques.

Oscillatory traction

The participant lies supine. Therapist with both hands holds occiput and performs an oscillatory occipital traction.

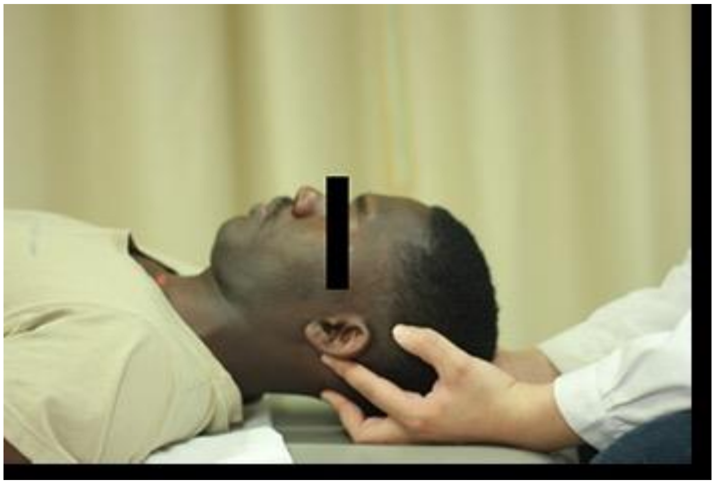

Craniocervical maintained traction

The participant lies supine, the therapist stands at the head of the plinth. The stabilizing hand holds C2 in position by placing the radial border of the index finger on the superior surface of the spinous process of C2. The mobilizing hand grips the occiput and applies the traction movement. 4 sets of 4 repetions of 10 seconds of sustained traction , followed by four seconds of rest between each repetition

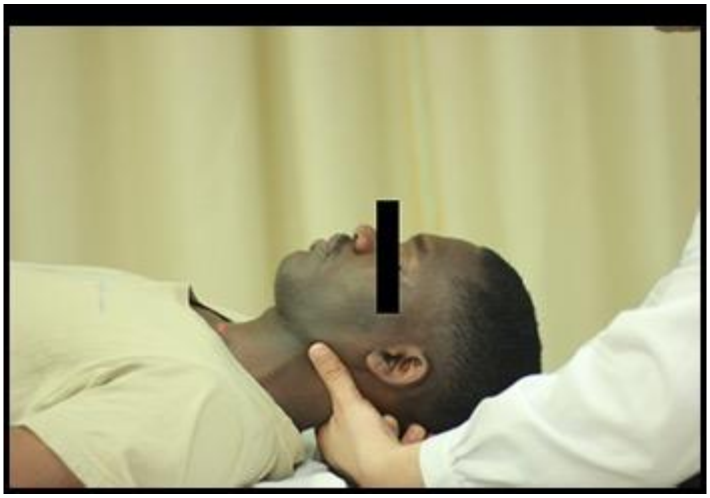

Upper cervical flexion mobilization.

The participant lies supine. The therapist places his stabilizing hand at the occiput and the mobilizing hand superior to the frontal bone. The mobilizing hand applies an anterior-caudal push while the stabilizing hand adds a light traction.

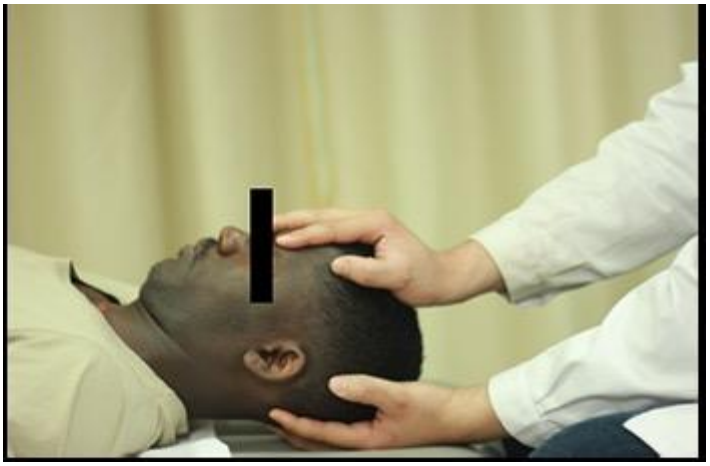

postero anterior mobilization from prone position for cervical spine by using thump finger:

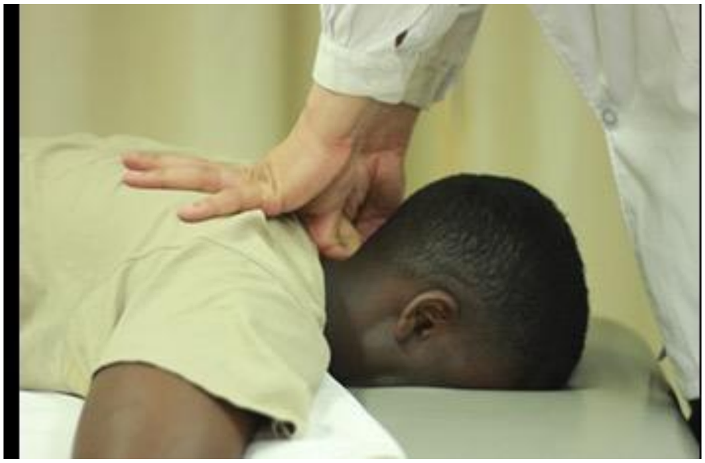

High-velocity technique in dorsal region.

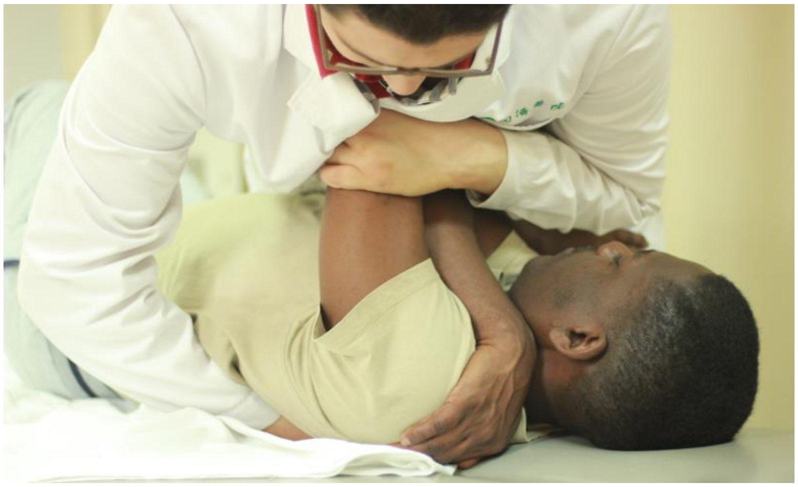

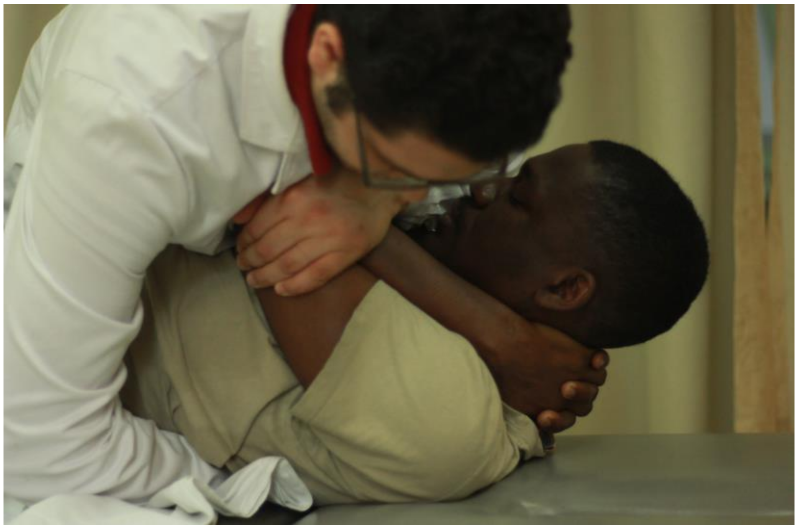

Retraction technique

The therapist places the wedge under the neck at C1-C2 level. Both hands stabilizing occiput and the shoulder placed superior to the frontal bone. The phases of the technique are: craniocervical traction and APUCM and upper cervical flexion mobilization.

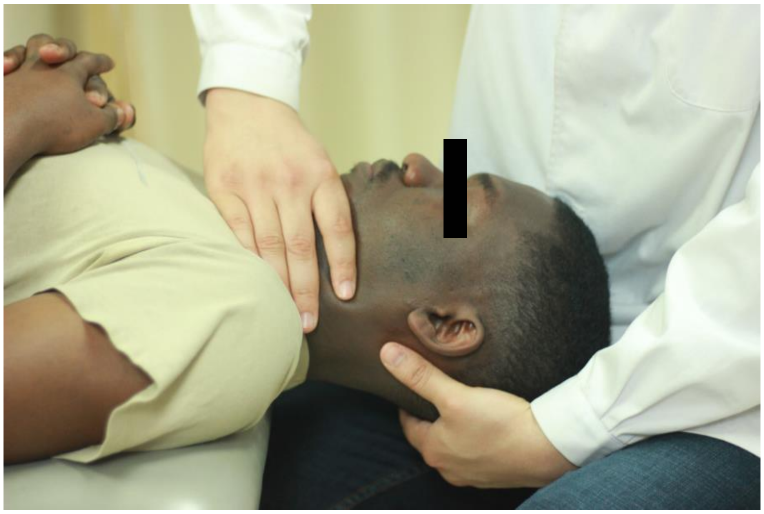

Therapeutic exercise:

According to the protocol described by Harman et al, (14), we will make therapeutic exercises program of the deep cervical flexors, shoulder retractors, and serratus anterior muscles.

Patients are explained to perform:

a) Five sets of isometric contraction of the deep neck flexors for 8-10 seconds.

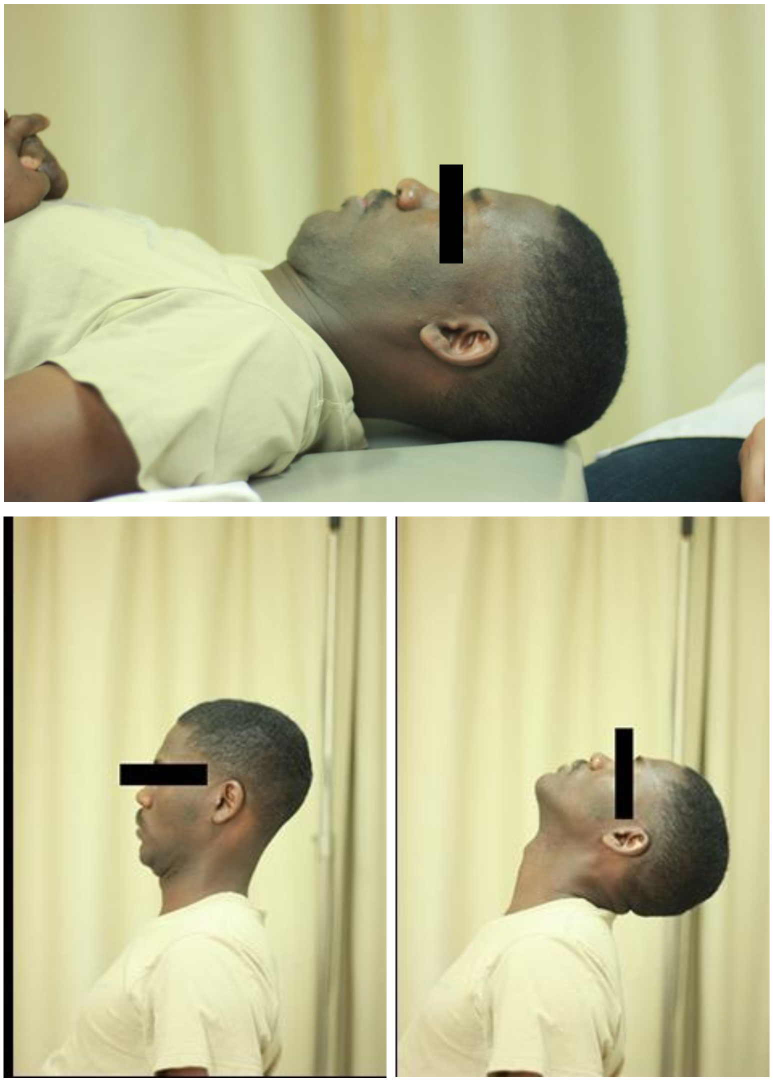

Stretch Cervical Extensors

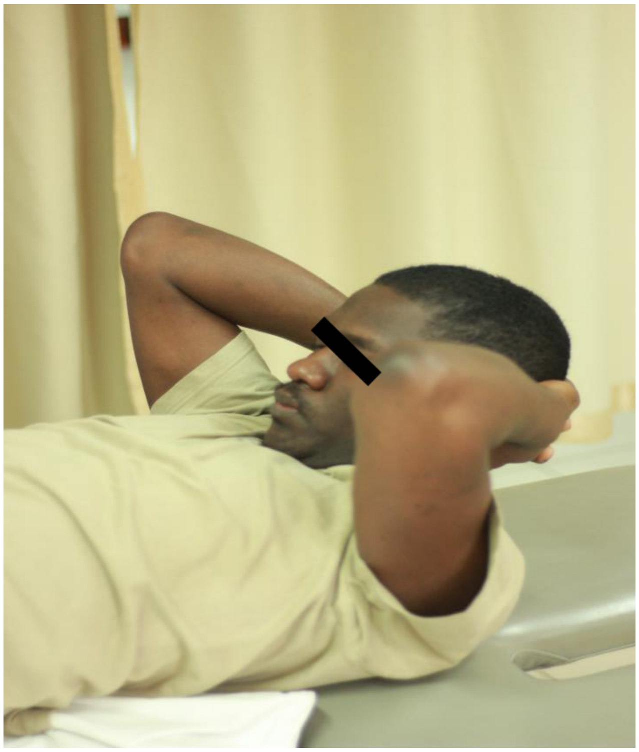

Strengthen Shoulder Retractors

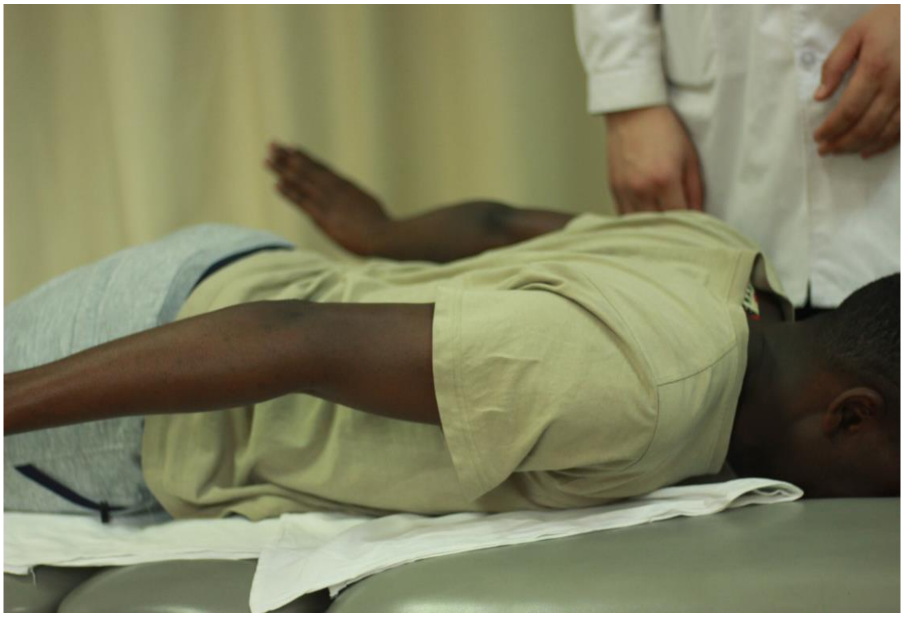

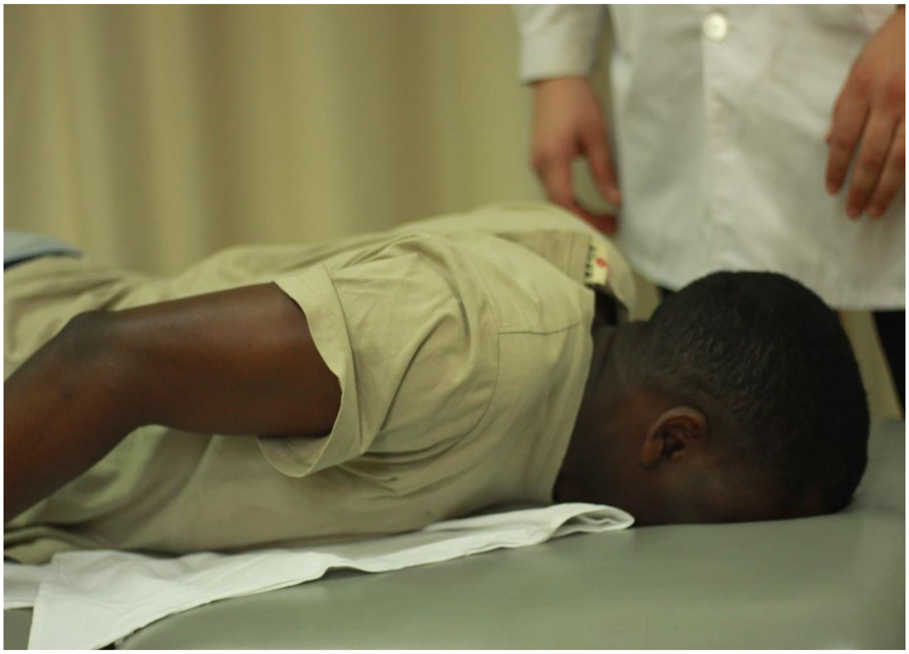

Stretch Pectoralis Muscle

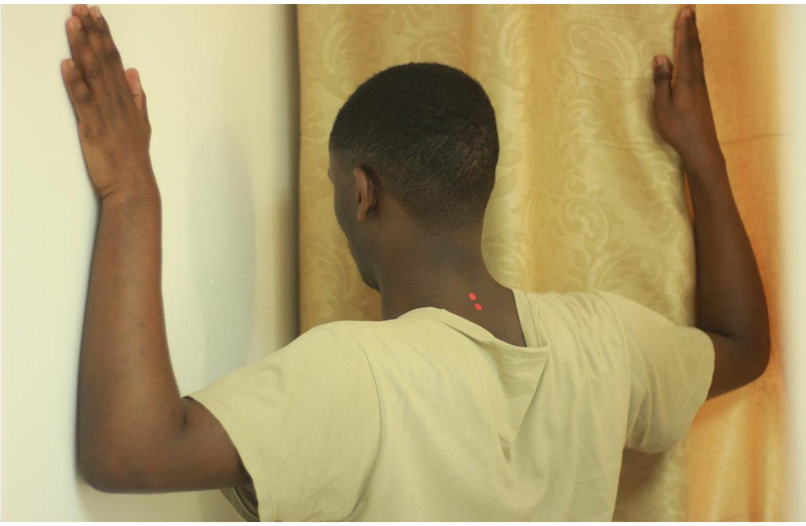

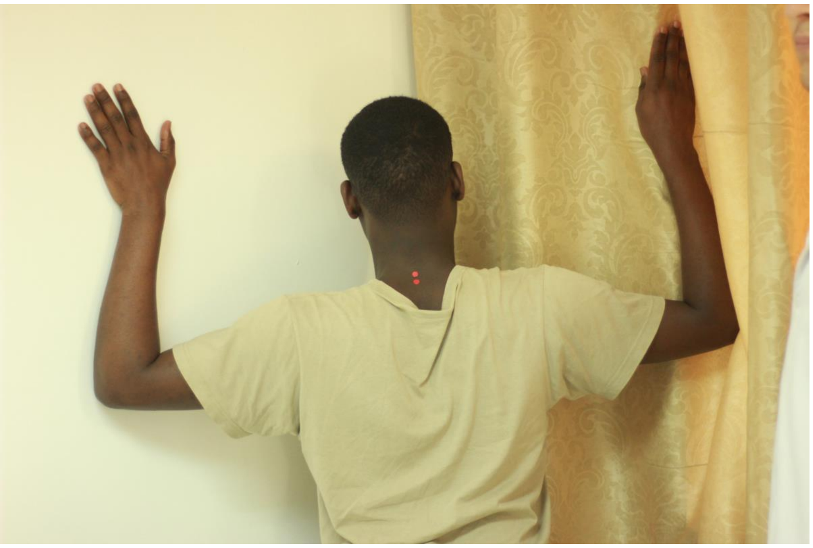

Supplement: Supplementary file 3 — Conventional treatment. (PDF 628 kb) [file 13063_2019_3337_MOESM2_ESM.pdf]
